# Supplementary material for: DMRforPairs: identifying Differentially Methylated Regions between unique samples using array based methylation profiles
Source: BMC Bioinformatics. 2014 May 15;15:141. doi: 10.1186/1471-2105-15-141 (PMC4046028; doi:10.1186/1471-2105-15-141)
Supplement: Additional file 2 — DMRforPairs output for the comparison of A431-MCF7 and NA17018-NA17105. Please start from the HTML files in each folder. Available via the BMC Bioinformatics website. [file 1471-2105-15-141-S2.zip › 1394847754114233_MOESM2_ESM/A431_MCF7/figures/40542.pdf]

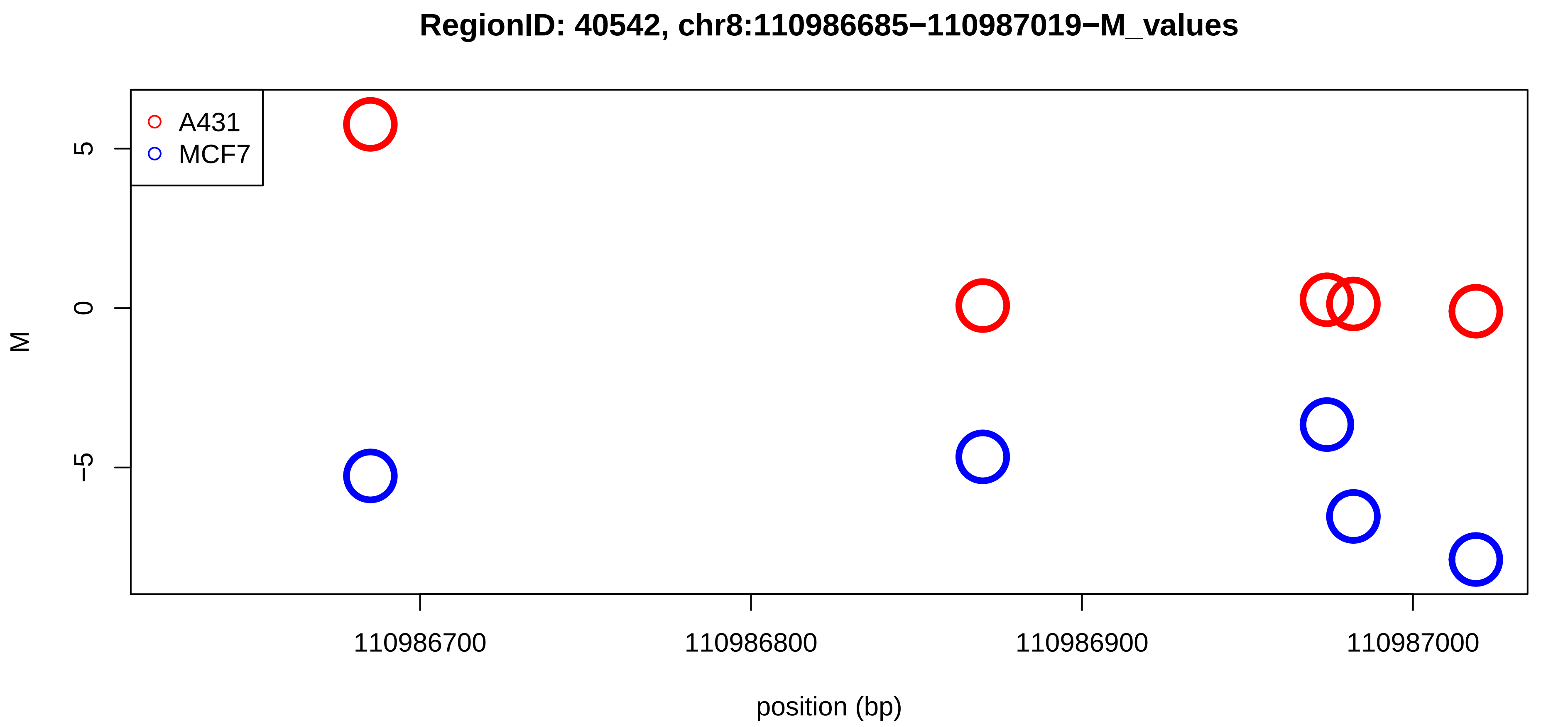

RegionID: 40542, chr8:110986685−110987019−M_values
A431
MCF7
M
5
0
−5
110986700
110986800
110986900
110987000
position (bp)

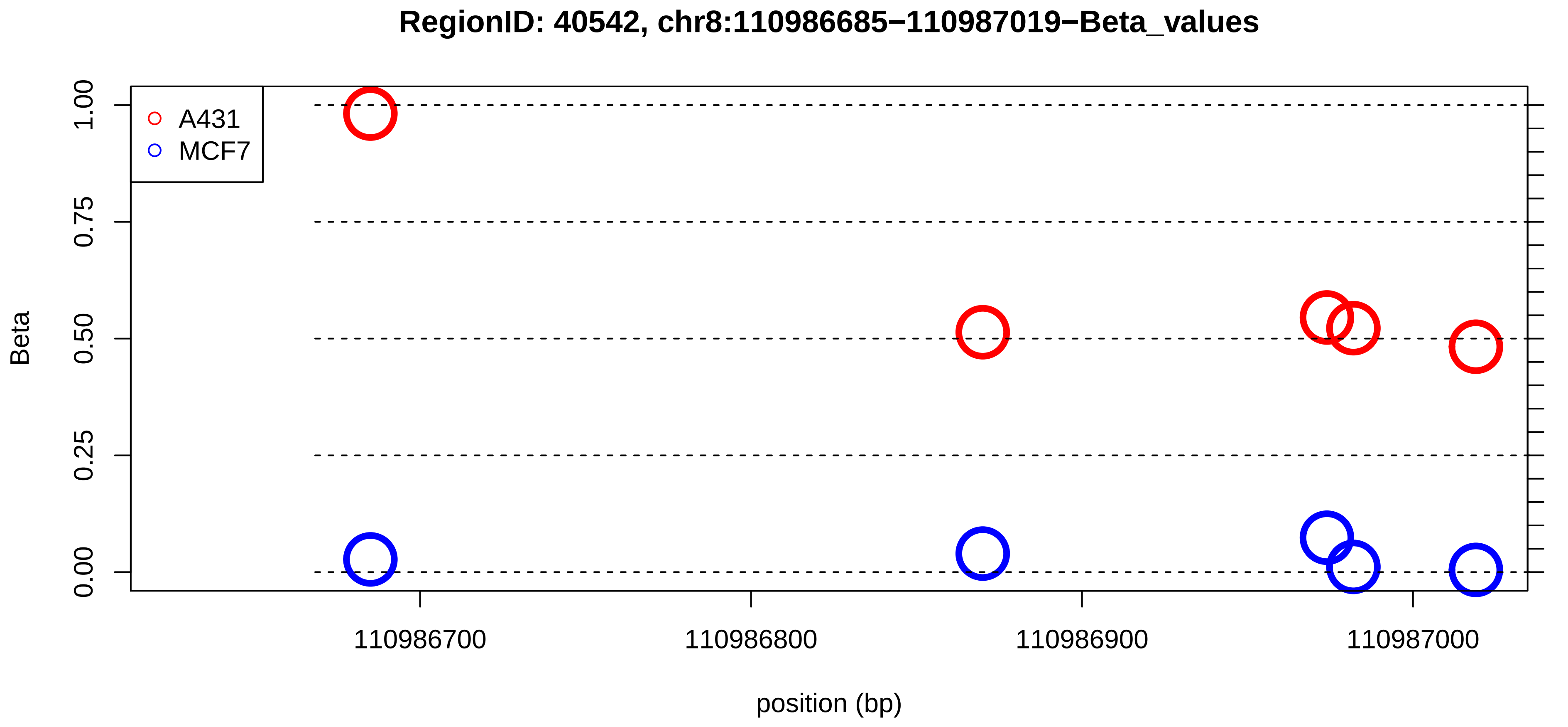

RegionID: 40542, chr8:110986685−110987019−Beta_values
A431
MCF7
Beta
1.00
0.75
0.50
0.25
0.00
110986700
110986800
110986900
110987000
position (bp)

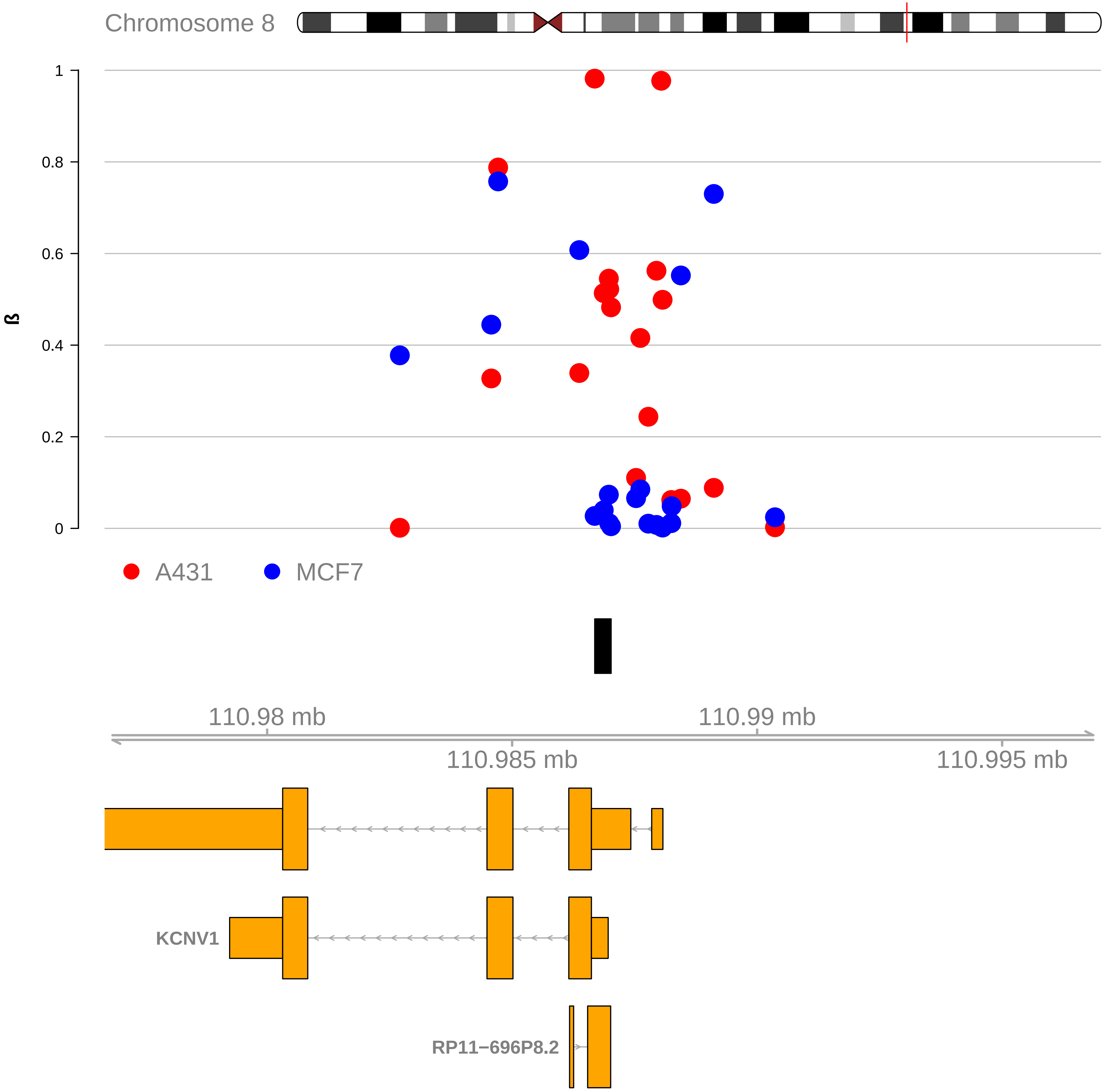
Chromosome 8
1
0.8
0.6
0.4
0.2
0
ß
A431
MCF7
110.98 mb
110.99 mb
110.985 mb
110.995 mb
KCNV1
RP11−696P8.2
